# Identification and Analysis of Genome-Wide SNPs Provide Insight into Signatures of Selection and Domestication in Channel Catfish (*Ictalurus punctatus*)

**DOI:** 10.1371/journal.pone.0109666

**Published:** 2014-10-14

**Authors:** Luyang Sun, Shikai Liu, Ruijia Wang, Yanliang Jiang, Yu Zhang, Jiaren Zhang, Lisui Bao, Ludmilla Kaltenboeck, Rex Dunham, Geoff Waldbieser, Zhanjiang Liu

**Affiliations:** 1 The Fish Molecular Genetics and Biotechnology Laboratory, Aquatic Genomics Unit, School of Fisheries, Aquaculture and Aquatic Sciences, and Program of Cell and Molecular Biosciences, Auburn University, Auburn, Alabama, United States of America; 2 USDA-ARS Warmwater Aquaculture Research Unit, Stoneville, Mississippi, United States of America; University of Iceland, Iceland

## Abstract

Domestication and selection for important performance traits can impact the genome, which is most often reflected by reduced heterozygosity in and surrounding genes related to traits affected by selection. In this study, analysis of the genomic impact caused by domestication and artificial selection was conducted by investigating the signatures of selection using single nucleotide polymorphisms (SNPs) in channel catfish (*Ictalurus punctatus*). A total of 8.4 million candidate SNPs were identified by using next generation sequencing. On average, the channel catfish genome harbors one SNP per 116 bp. Approximately 6.6 million, 5.3 million, 4.9 million, 7.1 million and 6.7 million SNPs were detected in the Marion, Thompson, USDA103, Hatchery strain, and wild population, respectively. The allele frequencies of 407,861 SNPs differed significantly between the domestic and wild populations. With these SNPs, 23 genomic regions with putative selective sweeps were identified that included 11 genes. Although the function for the majority of the genes remain unknown in catfish, several genes with known function related to aquaculture performance traits were included in the regions with selective sweeps. These included hypoxia-inducible factor 1β· *HIF*ι*β ¨* and the transporter gene ATP-binding cassette sub-family B member 5 (*ABCB5*). HIF1β· is important for response to hypoxia and tolerance to low oxygen levels is a critical aquaculture trait. The large numbers of SNPs identified from this study are valuable for the development of high-density SNP arrays for genetic and genomic studies of performance traits in catfish.

## Introduction

Strong selection can lead to significant allele frequency shifts with alleles directly affecting a trait reaching high frequencies. Due to genetic linkage, alleles nearby on the chromosome also change in frequency, leaving signatures of a selective sweep [1]. Selective sweeps have been detected in several agricultural animals such as chicken [2], [3], pig [4], and cattle [5], [6]. In addition to such hard selective sweeps where a single allele is selected for, recent work indicated that in some cases, more than one positive allele can be present within the selected loci, thus in this scenario drastic reduction in genetic variation in the genomic region does not occur. This type of selective sweep has been defined as soft selective sweep [7]–[9]. The lack of strong reduction in genetic diversity in regions with soft sweeps makes them more difficult to identify than hard sweeps. Therefore, the number of soft sweeps is likely underestimated [7].

In contrast, hard selective sweeps can be readily detected as they result in an increase of a specific allele, and hence, are more useful for the detection of genes underlining the performance traits. Studies of selective sweeps due to domestication in teleost fish are limited because most selective breeding of fish has been in the last 50 years, and there are few active selection programs for fish. Selective sweeps have been identified from fish species such as three-spined stickleback (*Gasterosteus aculeatus*) [10]–[12] and Atlantic salmon (*Salmo salar*) [13].

Domestication is one kind of selection involving the removal of some selection pressure typical of natural environments but intensification of others relevant to farming conditions [14]. For example, the anti-predator behavior of fish such as shoaling and schooling are essential for predator defense for wild fish [15], [16]. Under farm environments, there are either no or limited number of predators, and therefore the anti-predator behavior is no longer essential. Therefore, anti-predator behavior traits were reduced or totally lost in domesticated aquatic species such as rainbow trout (*Oncorhynchus mykiss*) [17] and laboratory strains of zebrafish (*Danio rerio*) [18], pumpkinseed sunfish (*Lepomis gibbosus*) [19], and brown trout (*Salmo trutta*) [20]. In rainbow trout, comparisons between individuals recently derived from wild stocks and domestic populations suggest significant genetic effects on mean swim level, hiding, foraging, startle response, and aggression level from domestication [21]. Similarly, Fine *et al.* [22] found that both spine and girdle exhibit negative allometric growth, and the pectoral spines and girdles are lighter in domesticated than in wild channel catfish (*Ictalurus punctatus*).

Genomic impact of domestication has not been well studied in fish species. Previous studies have shown morphological, behavioral and growth changes in channel catfish during domestication [22], [23], but the molecular basis of such changes has not been elucidated, due, at least in part, to the lack of molecular markers capable of providing whole genome coverage. In regards to domesticated channel catfish selected for body weight, significant changes in allozyme and microsatellite allele frequencies were found [24], [25].

Molecular markers have been developed from catfish [26], [27]. However, many types of markers such as microsatellites are well adapted to other genetic studies, but not particularly powerful in providing whole genome coverage for the analysis of selective sweeps, at least not with a high efficiency. Therefore, markers that allow whole genome coverage and high levels of automation must be developed for channel catfish. Despite some earlier efforts in developing single nucleotide polymorphism (SNPs) markers in catfish [27], large numbers of SNPs from intergenic regions of the genome are not available. We took advantage of the next generation sequencing to identify a large number of SNPs covering the genome of catfish. Through the use of populations used in aquaculture and a wild population, the next generation sequencing datasets allowed genomic analysis of regions with selective signatures. Here we report over eight million genomic SNPs and their application for the analysis of selective sweeps in channel catfish.

## Materials and Methods

### Fish sources and sampling

All procedures involving the handling and treatment of used fish during this study were approved by the Auburn University Institutional Animal Care and Use Committee (AU-IACUC) prior to initiation of the project. A total of 150 channel catfish, with 30 individuals from each of Marion, Thompson, USDA103, one outbred commercial strain (hereafter referred to as Hatchery), and one wild population were used for this study. The four aquaculture strains were from different geographic locations within the United States, which possess different production traits such as growth rate, disease resistance and feed conversion efficiency [28]. The Marion strain was originally from the Marion National Fish Hatchery, which provided stock for many of the catfish farms in Alabama [28]. The original fish for this strain were collected from the Red River, Arkansas, and other strains were later mixed with these fish. The Thompson strain was originally from Thompson-Anderson fingerling farms, which was one of the major fingerling farms in Mississippi. The origin of this strain can be traced primarily to the Yazoo River and to a lesser degree Red River and Kansas [28]. USDA103 was originally from US Department of Fish and Wildlife Hatchery in Uvalde, TX [29]. The Hatchery strain was originally from catfish farms in Mississippi, and was widely used in the catfish industry. The wild channel catfish used in this project were obtained from Coosa River, Alabama [30], [31].

### DNA extraction, library preparation and sequencing

The fish were euthanized with tricaine methanesulfonate (MS 222) at 300 mg/l before blood collection. For each individual, 500 µl blood was collected for DNA isolation, placed into 5 ml lysis buffer immediately, and then into a water bath at 55°C for 12 h. Total DNA was isolated using the DNeasy Blood & Tissue Kit (Qiagen, Valencia, CA, USA) following the manufacturer's protocol. Equal amounts of DNA (100 µg) from each individual were pooled for sequencing, one pool for each strain.

Sequencing was conducted commercially at HudsonAlpha Genomic Services Lab (Huntsville, AL, USA). Genomic libraries were prepared with the Paired-end Sequencing Sample Preparation Kit (Illumina, San Diego, CA) with 5 µg of genomic DNA for all strains, according to the manufacturer’s instructions. For each strain, the prepared DNA library was sequenced on one lane of the Illumina HiSeq 2000 platform for 100-bp paired-end reads. The short reads were deposited in the NCBI Sequence Read Archive (SRA) under Accession number SRA075234 (http://www.ncbi.nlm.nih.gov/sra).

### Reference mapping

Sequence mapping was performed using CLC Genomics Workbench (version 4.0.2; CLC bio, Aarhus, Denmark). Before mapping, raw sequence reads were trimmed to remove adaptor sequences, ambiguous nucleotides (N’s), extreme short reads (<30 bp) and low quality sequences (Quality score<20) using CLC Genomics Workbench. The quality of each sequence was assessed as follows: First, convert Q (base quality) was converted to an error probability (P):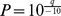. Then, for every base a new value was calculated for every base: *N* = P(A)-P(Q), where A is the criterion of the minimal quality score. In this project, A = 20 (Phred score); Q is the Phred quality score of each base. This value would be negative for bases with quality scores below 20. For every base, the software calculated the running sum of this value. The part of the sequence not trimmed will be the region between the first positive value of the running sum and the highest value of the running sum. Everything before and after this region was trimmed.

The clean reads from each strain were then aligned with the preliminary catfish genome assembly (unpublished data). The mapping parameters were set as: mismatch cost of 2, deletion cost of 3 and insertion cost of 3. The highest scoring matches that shared ≥95% similarity with the reference sequence across ≥90% of their length were included in the alignment. The mapping output was converted into BAM format [32] for further analysis.

### SNP identification and filtering

SNPs were identified from the pooled data from all the strains using the SAMtools (version 0.1.18) [32] and PoPoolation2 [33] with the lowest criteria setting to obtain all potential SNPs. Three factors that are important for excluding false SNPs caused by sequencing errors were set: 1) minimum read depth, 2) maximum read depth, and 3) minor allele read count. An optimal combination of these three factors was determined and used for screening quality SNPs. SNPs with the presence of both alleles in all five strains were defined as common SNPs. SNPs were defined as strain-specific SNPs if the SNP polymorphisms were found in only one strain. The information of identified SNPs were deposited in the National Animal Genome Research Program Aquaculture Genomics Data Repository (www.animalgenome.org/repository/pub/auburn2014.0530/).

### Analysis of Significant SNPs

SNPs with significantly different allele frequency ratios were identified between domestic catfish strains and the wild population (hereafter referred to as significant SNPs). Two-tailed Fisher’s exact test was performed with the statistical significance level of false discovery rate corrected P value ≤0.01. Significant SNPs were categorized into three groups based on their location: 1) in the coding regions, 2) near the coding regions and 3) on non-coding regions. Near the coding regions means the SNP is located on non-coding regions but within 100 bp from the coding regions.

### Selective sweep analysis

With the availability of significant SNPs, genomic regions with selective sweeps were identified from the four domestic strains by detecting the genome regions with extremely low heterozygosity. The pooled heterozygosity (*H*_p_) score was calculated using the formula *H*_p_ = 2Σ*n*_MAJ_Σ*n*_MIN_/(Σn_MAJ_ + Σ*n*_MIN_)^2^ [3], [4]. Σ*n*_MAJ_ was the sum of the major allele reads, and Σ*n*_MIN_ was the sum of the minor allele reads for all significant SNPs in one window. The *H*_p_ score was calculated based on 20 kb sliding window across the genome. Windows with less than five significant SNPs were not used for calculation. Putative selective sweeps were identified from windows with −log_2_(*H*_p_) score ≥4.

## Results

### Illumina sequencing and reference mapping

A total of 40.6–44.7 Gb of sequences were generated from each strain (Table 1). Approximately 96% reads were clean after trimming. The average lengths of the clean reads varied from 94 to 95 nucleotides. Reference mapping was conducted by aligning sequence reads from each strain with the preliminary catfish genome assembly (unpublished data). A total of 30.7–34.6 Gb were aligned to the reference sequences (Table 1). On average, around 31X–35X genome coverage (read depth) were obtained for each of the five populations. When all the sequences were combined, the total read depth was 167X genome coverage (Table 1).

**Table 1:** Summary of genomic data generation of channel catfish using Illumina HiSeq 2000, including raw data, trimmed reads, average length, reads mapped and genome coverage by strain.

Strains	Raw data	Trimmed reads	Average length	Reads mapped	Genome coverage
Hatchery	43.8 Gb	42.0 Gb	95.2 bp	32.6 Gb	33.3 X
USDA103	42.9 Gb	41.6 Gb	94.5 bp	33.7 Gb	34.4 X
Thompson	44.7 Gb	43.1 Gb	93.8 bp	34.6 Gb	35.3 X
Marion	42.3 Gb	40.8 Gb	94.2 bp	31.8 Gb	32.4 X
Wild population (Coosa River, AL)	40.6 Gb	39.3 Gb	94.8 bp	30.7 Gb	31.3 X
Total	214.3 Gb	206.8 Gb	94.5 bp	163.4 Gb	166.7 X

### Optimization of the *in-silico* identification of SNPs

**Figure 1:**
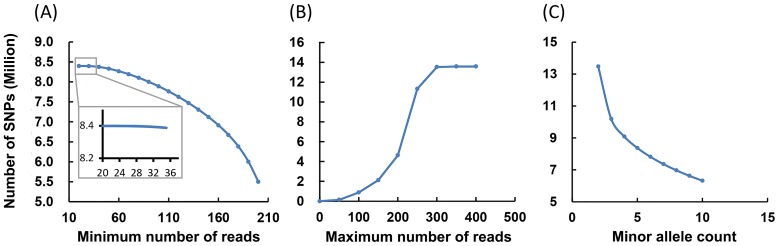
Influence of factors used for SNP filtering. (**A**) Influence of minimum reads on SNP identification. The x-axis represents the number of minimum reads used for SNP detection and the y-axis represents the number of SNP identified under a certain number of minimum reads. (**B**) Influence of maximum reads on SNP identification. The x-axis represents the number of maximum reads used for SNP detection and the y-axis represents the number of SNP identified under a certain number of maximum reads. (**C**) Influence of minor allele read counts on SNP identification. The x-axis represents the number of minor allele reads used for SNP detection and the y-axis represents the number of SNP identified under a certain number of minor allele reads.

To reduce false SNPs derived from sequencing errors, a set of criteria was first developed, including the minimum read depth, the maximum read depth and minor allele read count. As shown in Figure 1A, the impact of minimum read depth on SNP identification was tested in the 10–200 intervals with the increasing step of 10. Minimum read depth only had a small effect on the number of identified SNPs within the interval of 10–30. However, beyond this interval, the number of total SNPs was reduced gradually with the increase of minimum read depth (Figure 1A). Apparently, the greater the minimum read depth, the more reliable the SNPs are. However, the higher the minimum read depth, the fewer the reads that are qualified to be included in the analysis. A reasonable choice is to select the largest minimum read depth without significantly reducing the number of identified SNPs. Therefore, we set the minimum read depth at 30 for further analysis (Figure 1A).

Maximum read depth can have an impact on the quality of SNPs because extremely high numbers of reads are likely generated from non-unique sequences such as repetitive elements or paralogous sequences. Therefore, we evaluated the impact of maximum read depth on SNP identification. As shown in Figure 1B, the total numbers of SNPs did not increase significantly when setting the maximum read depth greater than 300. We then examined the contents of repetitive elements for the reads included in these read-depth intervals. As shown in Table 2, the contents of repetitive elements within each read-depth range were similar, up to the maximum reads of 300. However, the contents of repetitive element increased significantly when the maximum read depth were set greater than 300, indicating that a larger proportion of reads from retroelements and DNA transposons were included. To avoid the false SNPs caused by misalignment of reads from repetitive regions, we set the maximum read depth at 300 for further analysis.

**Table 2:** Summary of repetitive element analysis in the SNP flanking regions, including retroelements, DNA transposons and unclassified repetitive elements.

Coverage range	Retroelements	DNA transposons	Unclassified
50–100	29	82	7
100–150	34	69	6
150–200	29	89	3
200–250	28	74	4
250–300	46	80	2
>300	101	195	13

Numbers were expressed as the number of repetitive elements within 200,000 bp surrounding 1,000 SNPs (200-bp sequence for each SNP with 100 bp upstream and 100 bp downstream).

Minor allele frequency (MAF) not only affects the SNP applicability for future genetic studies because it directly determines the polymorphism information content of the SNP markers, but MAF also has an impact on the identification of quality SNPs. In general, the relationship curve can be arbitrarily divided into two phases: In the first phase, when minor allele counts were set as 2–4, the total number of SNPs was reduced sharply, while in the second phase, when minor allele reads were set as greater than 4, the total number of SNPs was also reduced, but at a much reduced rate, suggesting that minor allele reads of 4–6 may be appropriate for data in the present work (Figure 1C). Thus, the minor allele read counts were limited the minor allele read counts to be equal or greater than 5 for further analysis.

In addition to the initial assessment of these factors, the percentage of sequences that were included for SNP identification were examined. As shown in Table 3, the setting of minimum read depth and the minor allele read count did not have a major impact on the percentage of sequences included in the analysis. In contrast, the maximum read depth can have a drastic impact on the percentage of sequences to be included for analysis. For instance, when the maximum read depth was limited to 150 (note that average read depth of this study is 166.7 X), only 4.4% of sequences were included (Table 3). When the parameters were set at 30 for minimum read depth, 300 for maximum read depth, and 5 for minor allele read counts, almost 58% of sequences were included (Table 3). This set of criteria was used for the identification of quality SNPs, the analysis of strain-specific SNPs and the analysis of selective sweeps.

**Table 3:** Optimization of criteria for SNP identification in channel catfish, including minimum reads, maximum reads and minor allele count.

Criteria set	Minimum reads	Maximum reads	Minor allele count	% Reads included	Total SNP number
1	20	Excluding top 2%	2	100%	13,582,677
2	30	Excluding top 2%	2	74.7%	13,576,132
3	30	300	3	74.2%	10,217,482
4	30	150	3	6.4%	1,703,297
5	30	300	5	57.6%	8,395,720
6	30	150	5	4.4%	1,295,156
7	50	300	5	57.5%	8,329,404
8	50	150	5	4.4%	1,228,840

### SNP identification

A total of more than 13 million potential single nucleotide variations were observed at the most relaxed set of criteria, i.e., minimum read depth of 20, maximum read depth is set as excluding the top 2% of all reads, and minor allele read counts of 2. At our selected set of criteria, a total of 8,395,720 (∼8.4 million) putative SNPs (hereafter referred to as SNPs) were identified (Table 3).

**Figure 2:**
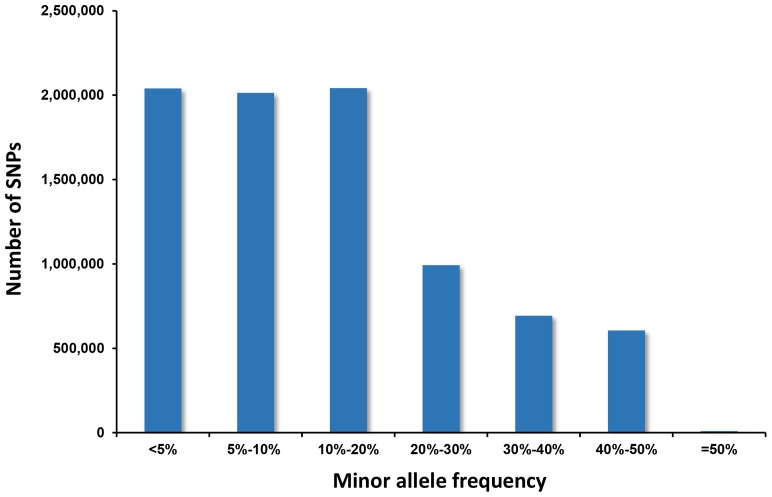
Distribution of SNP minor allele frequencies. SNPs were separated into six categories according to their MAF level. The first two categories contained the range of 5 percent and the other four categories contained the range of 10 percent.

These 8.4 million SNPs were subsequently used for the assessment of the distribution of minor allele frequencies. The MAF of each identified SNP was estimated based on the reference number and variant allele reads observed in the reference mapping. Approximately 4 million SNPs have an estimated MAF ≤10% (Figure 2). Over 4.3 million SNPs have an estimated MAF >10%, of which 2 million had a MAF of 10–20%; 992,502 had a MAF of 20–30%; 693,363 had a MAF of 30–40%; 606,046 had a MAF of 40–50%, and 9,305 SNPs had an equal minor and major frequencies at 0.5 (Figure 2).

### Identification SNPs within and among strains

Putative SNPs identified from each of the five strains were shown in Table 4. Overall, 7.1 million, 4.9 million, 5.3 million, 6.6 million and 6.7 million SNPs, were identified from the Hatchery strain, USDA103, Thompson strain, Marion strain, and wild population, respectively (Table 4). The largest numbers of SNPs were identified from the Hatchery strain, followed by Wild population, Marion strain, and Thompson strain. USDA103 was the strain with the least number of SNPs identified (Table 4).

**Table 4:** Summary of strain-SNPs in channel catfish, including quality SNPs in the strain, strain-specific SNPs and the percentage of strain-specific SNPs.

Strain	Quality SNPs	Putative strain-specific SNPs	Percentage
Hatchery	7,100,489	66,487	0.9%
USDA103	4,898,477	143,126	2.9%
Thompson	5,263,008	116,793	2.2%
Marion	6,569,112	88,251	1.3%
Wild (Coosa River, AL)	6,654,504	109,998	1.7%

SNPs that were observed from only one strain were considered as putative strain-specific SNPs. SNPs that were polymorphic in all strains were considered as common SNPs. Approximately, 2.7 million common SNPs were identified. The number of strain-specific SNPs identified from each of the five strains varied from 66,487 to 143,126, accounting for 0.9%, 2.9%, 2.2%, 1.3%, and 1.7% of SNPs that were identified from that strain, respectively (Table 4).

### Analysis of selective sweeps

As shown in Table 5, a total of 407,861 significant SNPs were identified, which had significant differences in allele frequencies between domestic strains and the wild population (Fisher’s exact test, FDR p-value ≤0.01). Of these 407,861 significant SNPs, 52,076 were located in coding regions, 21,232 were located within 100 bp of coding regions, and 334,553 were located in non-coding regions.

**Table 5:** Summary of SNPs with significant differences in allele frequencies between four domesticated strains and one wild population in channel catfish.

Category	SNP number
Significant SNPs	407,861
Significant SNPs in coding regions	52,076
Significant SNPs near coding regions	21,232
Significant SNPs in non-coding regions	334,553

**Figure 3:**
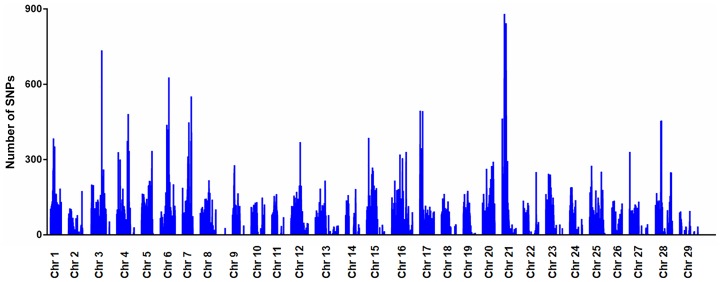
Genome-wide distribution of significant SNPs. Physical positions of all catfish 29 chromosomes are presented on the x-axis, and significant SNP numbers within a window size of 200 Kb is given on the y axis.

A total of 237,655 (58.3%) significant SNPs were assigned to 29 tentative chromosomes based on the catfish linkage map [34]. The distribution of significant SNPs within chromosomes with the number of significant SNPs in 200 kb bins across each chromosome is illustrated in Figure 3. All of the 29 catfish chromosomes contained significant SNPs, with chromosome 3, chromosome 6 and chromosome 21 harboring the largest number of significant SNPs (12,494, 12,417 and 12,340, respectively). Chromosome 29 contained the least number of significant SNPs (1,717). Regions with the largest number of significant SNPs were from chromosome 21.

**Figure 4:**
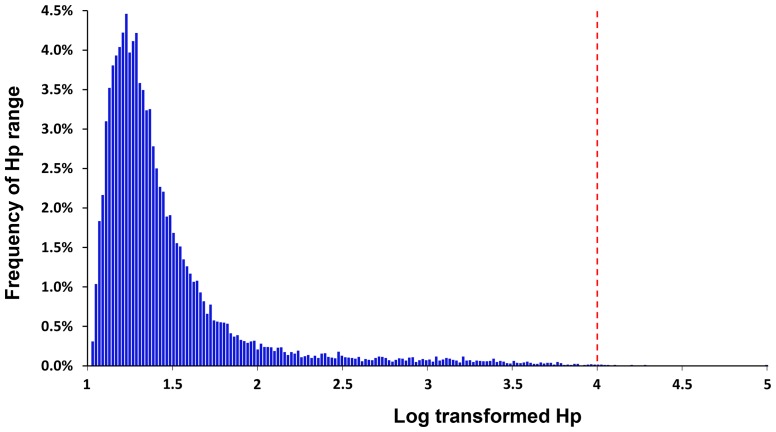
Histogram of log-transformed pooled heterozygosity (*Hp*) values. The x-axis is evenly divided into 200 bars from 1 to 5, and each bar represents a transformed *Hp* range of 0.02. The y-axis represents the percentage of each transformed *Hp* range in the total 200 transformed *Hp* ranges. All *Hp* values were transformed by –log_2_.

Analysis for selective sweeps was performed as described by Rubin et al. [3], [4]. The pooled heterozygosity (*H_p_*) was calculated in 20-kb windows based on the major and minor alleles of significant SNPs, and were then log transformed. Most of the windows (73.5%) had the log-transformed *H_p_* scores between 1 and 1.5, indicating high levels of heterozygosity (Figure 4). A total of 23 windows (0.1%) with log-transformed *H_p_* score ≥4, indicating excessive levels of homozygosity in these regions, were identified as genomic regions with putative selective sweeps (Table S1).

**Figure 5:**
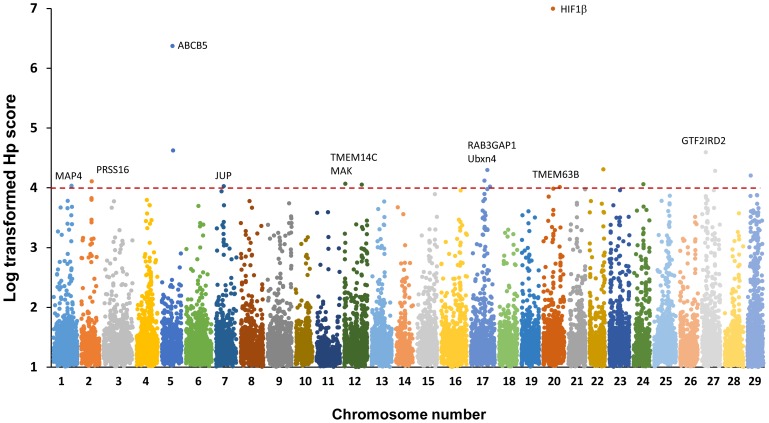
Genome-wide distribution of log-transformed pooled heterozygosity (*Hp*) values. The x-axis represents the positions of windows (20 Kb) along each chromosome, which is represented with different colors. The y-axis represents the *Hp* scores transformed by –log_2_. Windows of *HIF-1β* had the *Hp* score of 0, therefore, its transformed *Hp* score was defined as 7, the maximum score, for the convenience of plotting.

The distribution of the 23 regions with selective sweeps in catfish genome was then analyzed. As shown in Figure 5, these regions were distributed among different chromosomes. Among them, chromosome 5, 12, 17 and 20 contained more than one region with selective sweeps. Chromosome 20 contained a region with the lowest level of heterozygosity. The *H_p_* score of this region was 0 and therefore the log-transformed *H_p_* score was infinite. Thus, a value of 7 was assigned, which was the highest log transformed *H_p_* score (Figure 5) for the convenience of plotting.

A total of 11 genes were found from these genomic regions with selective sweeps (Table 6). These genes were located on eight chromosomes including chromosome 1, 3, 5, 7, 12, 17, 20 and 27. Among these genes, hypoxia-inducible factor 1-beta (*HIF-1β)* had the most significant *H_p_* score, which was followed by ATP-binding cassette sub-family B member 5 (*ABCB5*).

**Table 6:** List of genes identified from the regions with selective sweeps and their chromosomal location, pooled heterozygosity score and putative functions for channel catfish.

Chromosome	Pooled heterozygosity	Log-transformed *Hp*	Gene name	Putative function
Chr 20	0	**–**	*HIF-1β*	Stress response
Chr 5	0.012	6.38	*ABCB5*	Unknown
Chr 17	0.051	4.30	*RAB3GAP1*	Eye/brain development
Chr 17	0.051	4.30	*Ubxn4*	ERAD
Chr 27	0.051	4.30	*GTF2IRD2*	Transcription factor
Chr 3	0.058	4.11	*PRSS16*	T cell development
Chr 12	0.060	4.07	*TMEM14C,*	Heme biosynthesis
Chr 12	0.060	4.07	*MAK*	Spermatogenesis
Chr 1	0.061	4.03	*MAP4,*	Microtuble assembly
Chr 7	0.061	4.03	*JUP*	Junctional plaque protein
Chr 20	0.062	4.01	*TMEM63B*	Unknown

## Discussion

In this study, next generation sequencing was conducted for multiple individuals from four aquaculture strains and one wild population to identify SNPs for determination of genomic impact of domestication. The large numbers of SNPs identified from this study will be useful for the development of high density SNP arrays for genetic and genomic analysis in catfish [35].

Pooled sequencing has been utilized as an efficient and reliable approach for detecting and genotyping SNPs from populations [36]. One of the challenges for this approach is to distinguish the real from false SNPs. Validation of millions of SNPs is not practical and extremely costly if not impossible. Strategies to increase SNP conversion rate need to be developed. To increase the likelihood for the identification of real SNPs, major factors affecting SNP identification need to be assessed, of which, the maximum reads, minimum reads and minor allele read count were the most important and common factors, incorporated into various SNP detection tools [32], [33], [37], [38].

In that regards, reasonable criteria for SNP identification were set at a minimum read depth of 30, maximum read depth of 300, and minor allele count of 5, and 8.4 million putative SNPs were identified from five different catfish strains. On average, there was one SNP every 116 bp in channel catfish genome. Approximately, 66,000–143,000 SNPs were identified as strain-specific for each strain (Table 4), which in total account for approximately 6% of all SNPs. If more strains were evaluated than the 5 in this study, the proportion of strain-specific SNPs would likely be reduced. Catfish strains are almost impossible to distinguish based on phenotypes [29], therefore, these SNPs can be potentially used for strain identification, tracing the origin of commercial strains, and analyzing the genetic difference among strains and to mark fish for other genetic experiments. The 2.7 million common SNPs that are polymorphic in all five catfish populations will provide the main resources for SNP array design [35] and high-density linkage map development.

Liu *et al.* [27] sequenced 48 individuals of channel catfish from different strains (Marion, Pearson, Moyer, Holland and Noble) using pooled samples and detected more than two million putative gene-associated SNPs with more than 0.5 million being high quality SNPs. Approximately, 66% (341,663) of the high quality SNPs were identified in our results, supporting the confidence of parameters used in this project. The remaining 34% of SNPs that were not shared by these two studies may be caused by the use of different strains, as well as the relatively stringent parameters used for SNPs calling in this study.

SNPs with significant differences in allele frequency between domestic and wild catfish populations were identified to provide insight into genomic impact of domestication and selection. Compared with all the SNPs identified from channel catfish, significant SNPs were approximately 5% of the total SNPs, indicating that the vast majority of genomic regions have not been affected by domestication or selection. Additional analysis was conducted to determine the position and genes associated with significant SNPs. The vast majority of significant SNPs (87.2%) were located in the non-coding DNA sequences, while 12.8% of the significant SNPs were found in coding regions of catfish genes. This proportion of SNPs associated with genes is greater than the proportion of gene sequences from the whole genome sequences, suggesting that domestication and selection may have had a greater impact on genes than on intergenic regions.

The significant SNPs were distributed on each of the catfish chromosomes (Figure 3). Chromosome 3, 6 and 21 contained a largest number of significant SNPs, but from which no putative selective sweeps were identified. Perhaps, the catfish genome harbors a large amount of genetic variation for further domestication and selective breeding given the relatively short domestication and history of selection. Also, recent studies indicate that soft sweeps are abundant in adaptation and may play a major role in the rapid adaptation in many species [7]. Because soft sweeps contain multiple adaptive alleles and they all have relatively high frequencies, their genetic diversities should also be high. In this project, we only focused on hard selective sweeps from pooled sequencing data by searching the regions with low genetic diversity. Soft sweeps may be present in those chromosomes with abundant SNPs, but we only conducted our analysis with bi-allelic SNPs and our analysis does not provide any insight into soft sweeps.

A concern regarding the analysis of channel catfish was sampling since this species occupies a large geographical range, populations can be large and numerous domestic and wild populations exist. Assuming that all domesticated populations and a broad representation of wild populations can be achieved, significant SNPs between the domestic and wild populations could be used to reveal solid selective sweeps caused by domestication and selection. However, based on the nature of catfish industry, it is difficult to sequence large enough samples that can represent all genetic variations that exist in all domestic and wild strains. Therefore, we fully acknowledge the difficulties involved in the sampling of the domestic and wild populations for an aquatic species, however, analysis of putative selective sweeps should still provide insights into the potential impact of domestication on genome evolution. To identify hard type selective sweeps in domestic catfish caused by selective breeding, we analyzed the pooled heterozygosity (*H_p_* scores) for the domestic populations using significant SNPs with the assumption that artificial selection by domestication tends to create runs of homozygosity [39].

When hard selective sweeps are analyzed using the method of Rubin et al [3], [4], two parameters could affect its accuracy and sensitivity. The first is the window size used for the calculation of *H_p_* scores. Large window sizes could contain more SNPs and reduce the bias in the calculation of pooled heterozygosity, but it will also lose sensitivity due to the uneven distribution of SNPs. In catfish, where the whole genome has not been fully assembled, the window size should be set smaller than those species with whole genome reference assemblies simply because very long contigs are not yet available. After reviewing variable window sizes, we used 20-kb siding windows. Another noteworthy parameter is the SNP number in each window. Obviously, windows with very small SNP number cannot provide the actual heterozygosity of the genome regions they represent. Therefore, we did not include the windows that contained less than five significant SNPs in the analyses.

Domestication and selection could change genetic variability, the genetic correlations among traits and the interactions among loci. Traits with high production values, such as growth rate, disease resistance and tolerance to low oxygen have been selected for generations in aquaculture species either intentionally or unintentionally. Resistance to low oxygen is an important aquaculture trait relevant not only for survival, but also growth and disease resistance. Hypoxia can cause high mortality for aquaculture species. Even if the fish survive under hypoxic conditions, exposures to low oxygen levels often trigger disease incidents that cause further major losses [40], [41]. Variations in tolerance for low oxygen have been well studied with various aquaculture species [42]–[44]. However, genetic variation for low oxygen tolerance have not been systematically determined. In case of catfish, great efforts have been made on the genetic improvement of the important production traits, such as growth rates, disease resistance, tolerance to handling stress and hypoxia [45]–[47], but little is known of the genomic basis for such observed phenotypic improvements.

In the current study, a total of 23 genomic regions were identified that contained the signature of selective sweeps (log transformed *H_p_* score >4, Table S1), which could be the strong candidates for further studies of domestication in channel catfish. These 23 regions were located in different chromosomes (Figure 5), suggesting that multiple traits or multiple loci controlling a few traits could have responded to domestication. A selective sweep caused by domestication was identified in channel catfish Chromosome 17 (Pooled heterozygosity = 0.051), which is highly homologous to zebrafish Chromosome 9 [48]. A QTL responsible for the anti-predator behavior on zebrafish Chromosome 9 was detected by three different measures [18]. However, since those genomic regions are still large, it is not certain if the same genomic regions were under selection in zebrafish and in catfish. In three-spined stickleback, analysis for selective sweeps was conducted between ancestral oceanic populations and newly established freshwater populations [12]. A total of nine regions were identified with adaptive significance, three of which were supported by the previous QTL analysis on fresh water adaption. Domesticated strains and wild populations of in Atlantic salmon were compared using 261 SNP and 70 microsatellite markers [13]. A total of ten genomic regions were identified from different chromosomes with 14 genes identified from these regions. However, there was no overlap between these genes with our findings in channel catfish.

In the present study, we identified 11 genes from 9 of the 23 genomic regions with selective sweeps (Table 6 and Table S1). Two genes, hypoxia-inducible factor-1-beta (*HIF-1β*) and ATP-Binding Cassette, Sub-Family B, Member 5 (*ABCB5*), were located in the first two strongest hard sweeps (Figure 5). *HIF-1β* was located on the selective sweep region with *H_p_* = 0, meaning that all the significant SNPs located in this region were homozygous in all domestic populations and were heterozygous in the wild population. HIF-1β, also referred to as Aryl hydrocarbon receptor nuclear translocator (ARNT), mediates aryl hydrocarbon signaling and facilitates gene activation by dimerization with aryl hydrocarbon receptor (AHR) [49]. It is involved in the hypoxia response pathway where it forms heterodimers with HIF-1α, which in turn binds to P300 to activate a variety of hypoxia-responsive genes upon exposure to hypoxia [50], [51]. It is reasonable to conclude that selection for hypoxia tolerance under aquaculture conditions could have had a major genomic impact in this genomic region.

*ABCB5* is a member of ATP-Binding Cassette transporter gene family and only exists in vertebrates [52], [53]. It is highly expressed in melanocytes and may play an important role in melanomagenesis [52], [54]. The expression of *ABCB5* was also significantly associated with tumor progression and recurrence, acting as an energy-dependent drug efflux transporter and function during the multidrug resistance process [55], [56]. Studies on childhood obesity reported a CNV region on *ABCB5* gene that was exclusively associated with childhood obesity [57]. In our results, *ABCB5* was located in the second strongest selective sweep region (Table 6), suggesting extremely low genetic diversity block around the genomic region containing the *ABCB5* gene.

Several other genes such as UBX domain protein 4 and General transcription factor II-I repeat domain-containing protein 2A were also identified within the genomic regions with selective sweeps (Table 6). However, their roles in domestication are unknown at present and warrant future studies.

Considering the smaller effective population size of domestic strains at research institutions compared to than wild populations, some random genetic changes may take place due to founder effect and genetic drift. However, commercial populations are much larger than wild populations, but still could be impacted by founder effects. These would be partially offset by crossbreeding as many commercial populations originated from multiple strains [28]. Our findings of domestication related regions and genes could provide some insights into the genetic explanation of the differences between domestic and wild channel catfish in performance, morphology and behavior traits. For instance, the smallest numbers of SNPs were detected in USDA103. This may have been a result of historically small population sizes, founder effects from one or more brood stock transfers between hatcheries and research institutions, and intense selection for growth as this was one of the fastest growing domestic strains even before the recent directed selection [45]. Additionally, a large number of SNPs identified in this project using stringent criteria have been included in the construction of catfish SNP array [35] and will be further utilized in analysis of population diversity, development of high-density linkage maps and genome-wide selection.

## Supporting Information

Table S1 **Summary of the 23 genomic regions with selective sweeps, including channel catfish scaffold ID, window number, pooled heterozygosity score, scaffold position and the genes located in the regions.**
(XLSX)
